# Home-based, early intervention with mechatronic toys for preterm infants at risk of neurodevelopmental disorders (CARETOY): a RCT protocol

**DOI:** 10.1186/1471-2431-14-268

**Published:** 2014-10-15

**Authors:** Giuseppina Sgandurra, Laura Bartalena, Giovanni Cioni, Gorm Greisen, Anna Herskind, Emanuela Inguaggiato, Jakob Lorentzen, Jens Bo Nielsen, Elisa Sicola, the CareToy Consortium

**Affiliations:** Department of Developmental Neuroscience, IRCCS Fondazione Stella Maris, Viale del Tirreno 331, 56128 Calambrone Pisa, Italy; Neonatal Intensive Care Unit, Pisa University Hospital “Santa Chiara”, Via Roma 67, 56126 Pisa, Italy; Department of Clinical and Experimental Medicine, University of Pisa, Via Roma, 56125 Pisa, Italy; Department of Neonatology, Copenhagen University Hospital (Rigshospitalet), Copenhagen, Denmark; Department of Neuroscience and Pharmacology, University of Copenhagen, Copenhagen, Denmark; Helene Elsass Center, Charlottenlund, Denmark; Scuola Superiore Sant’Anna, Institute of Life of Sciences, Piazza Martiri delle Libertà 1, 56127 Pisa, Italy; Department of Nutrition, Exercise and Sport, University of Copenhagen, Copenhagen, Denmark

**Keywords:** Early intervention, Preterm infants, RCT, Tele-rehabilitation, ICT, Medical bioengineering, Neurodevelopmental disorders

## Abstract

**Background:**

Preterm infants are at risk for neurodevelopmental disorders, including motor, cognitive or behavioural problems, which may potentially be modified by early intervention. The EU CareToy Project Consortium (http://www.caretoy.eu) has developed a new modular system for intensive, individualized, home-based and family-centred early intervention, managed remotely by rehabilitation staff. A randomised controlled trial (RCT) has been designed to evaluate the efficacy of CareToy training in a first sample of low-risk preterm infants.

**Methods/Design:**

The trial, randomised, multi-center, evaluator-blinded, parallel group controlled, is designed according to CONSORT Statement. Eligible subjects are infants born preterm without major complications, aged 3-9 months of corrected age with specific gross-motor abilities defined by Ages & Stages Questionnaire scores. Recruited infants, whose parents will sign a written informed consent for participation, will be randomized in CareToy training and control groups at baseline (T0). CareToy group will perform four weeks of personalized activities with the CareToy system, customized by the rehabilitation staff. The control group will continue standard care. Infant Motor Profile Scale is the primary outcome measure and a total sample size of 40 infants has been established. Bayley-Cognitive subscale, Alberta Infants Motor Scale and Teller Acuity Cards are secondary outcome measures. All measurements will be performed at T0 and at the end of training/control period (T1). For ethical reasons, after this first phase infants enrolled in the control group will perform the CareToy training, while the training group will continue standard care. At the end of open phase (T2) all infants will be assessed as at T1. Further assessment will be performed at 18 months corrected age (T3) to evaluate the long-term effects on neurodevelopmental outcome. Caregivers and rehabilitation staff will not be blinded whereas all the clinical assessments will be performed, videotaped and scored by blind assessors. The trial is ongoing and it is expected to be completed by April 2015.

**Discussion:**

This paper describes RCT methodology to evaluate CareToy as a new tool for early intervention in preterm infants, first contribution to test this new type of system. It presents background, hypotheses, outcome measures and trial methodology.

**Trial registration:**

ClinicalTrials.gov: NCT01990183. EU grant ICT-2011.5.1-287932.

## Background

The incidence of preterm births has increased and survival rates in very preterm infants have improved over the past two decades [1, 2]. However, a significant number of these infants, especially if born with a very low birth weight (VLBW), show a developmental disorder. Follow-up studies indicate that up to 15% of infants are diagnosed with cerebral palsy and about 50% show cognitive, motor, or behavioural problems in childhood [3–5], resulting in a lower high school graduation rate compared with infants born at term with normal birth weight [6].

Early developmental interventions have been used in the clinical setting with the aim of improving the overall outcome for preterm infants [7]. Early Intervention (EI) means intervening as soon as possible to tackle problems that have already emerged due to perinatal or congenital brain disorders. It is carried out in a critical period of development (i.e. a time window during which specific functions develop very rapidly), when initial signs of atypical development are present but before they become overt. The target of EI training is to strengthen brain plasticity, higher in the first years of life, and to allow a better functional outcome. According to the neuroscience evidences, the activity-dependent neural plasticity is able to mould the neural development mainly during the critical periods, as indicated by several examples especially for motor and visual systems, both in animal models and in infants with brain lesion [8–12].

Based on our current neurobiological understanding, EI programs have to be implemented very early in life and should be intensive, repetitive, incrementally challenging, and individualized. They should include also goal-directed components and exercises where meaningful goals are provided, to give opportunities for problem solving and to indirectly drive the movements required to successfully meet task demands [13, 14]. The therapeutic approach should also involve repeated practice of tasks, which the infant wants to do [15]. There is also accumulating evidence of the importance of an enriched environment (EE) on infant's neurodevelopment [16–20]. Home EE should be organized to encourage the infant to perform specific tasks, tailored on the developmental needs of the individual infant, in an environment where the parent is actively and positively engaged with the child, to facilitate and promote learning. The home environment should include safe toys appropriate to the infant's ability level but posing a learning challenge and family interactions. In this context, as proposed by Morgan et al. [21], EE intervention could be defined as a set of modifications aimed to enrich motor, cognitive, sensory, or social aspects of the infant’s environment with the purpose of promoting learning.

Systematic and Cochrane Reviews [5, 22, 23] indicate an evidence of the positive effects of EI, mainly based on parents involvement, on the development of cognitive and motor functions in preterm infants. However, the identification of the most effective intervention strategies is difficult and more RCT, including control groups, with well defined content, focus, duration and intensity are deeply needed.

Moreover, providing infants at high risk of neurodevelopmental disorders by an EI personalized and with significant intensity and duration would impose high costs for the Health Care System. Even in western countries resources are unlikely to be sufficient to cover all needs. Moreover, some families have difficulties, for different reasons, to reach health care services. Biotechnologies, tele-rehabilitation and eHealth could represent a promising approach to provide home EI programs for a large number of infants at a relatively low cost.

**Figure 1:**
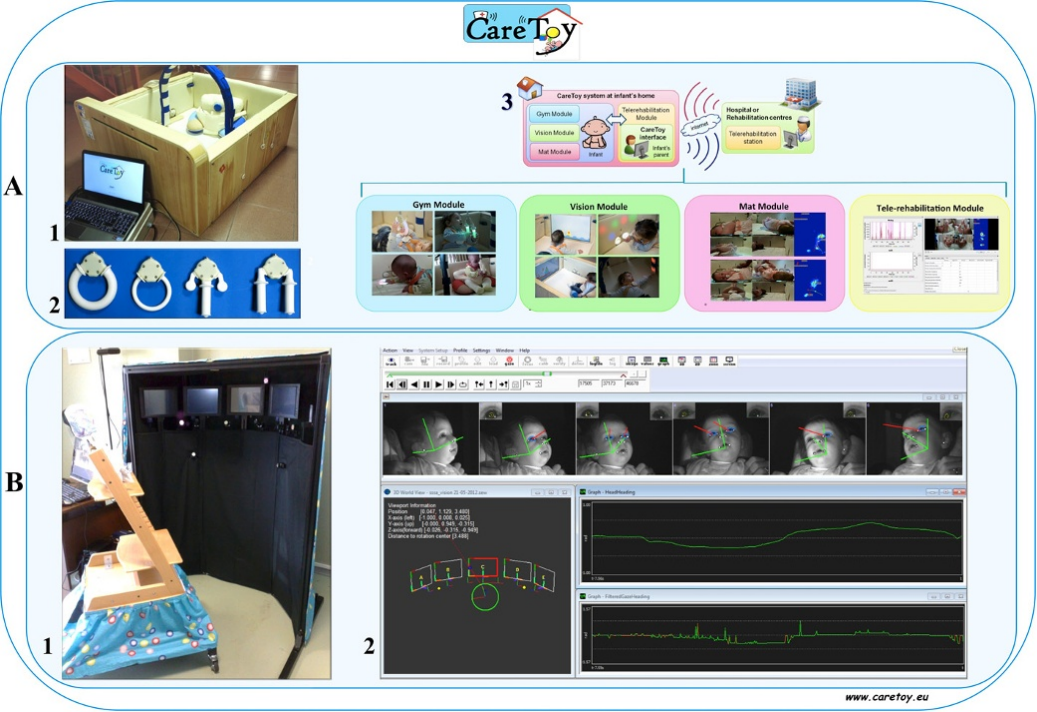
**CareToy for Home and CareToy for Clinics. A**, 1) Set up of CareToy for Home (*CareToy H*). It is delivered at home and used to promote infants’ motor and perceptual development, through individualized goal directed activities (rehabilitation packages of CareToy training). 2) Kit of sensorized toys. 3) The four modules of CareToy H (Gym, Vision, Mat and Tele-rehabilitation). **B**, 1) CareToy for Clinics (*CareToy C*), installed at the rehabilitation centres. 2) Reconstruction of the gaze of an infant during the observation tasks.

On this background, the CareToy Project (http://www.caretoy.eu, Trial Registration: NCT01990183) has aimed to develop a new technological smart modular system as a tele-rehabilitation tool for EI in infants at risk for neurodevelopmental disorder. The CareToy system for EI, called CareToy Home is composed by four modules: 1) *gym module* made of i) a kit of sensorized toys with different shape and size, ii) two interactive walls (feedback wall with embedded sounds and lights), iii) a belt wall with a sensorized pillow, iv) an arch with lights, v) 4 cameras; 2) *vision module* (screen wall), equipped with a large monitor; 3) *mat module* with a sensorized mat and three wearable sensors (two bracelets and chest strap) [24, 25] and 4) *tele-rehabilitation module* that allows the communication between CareToy system and clinical centre (Figure 1A).

The software includes principles, procedures and computer algorithms for data acquisition, processing and sensory integration from various sensors. Signal processing is performed in two different modes: i) real-time processing that provides information relevant for generating immediate feedback to the infant based on his/her activity and ii) post-processing of data that allows more detailed off-line analysis of the infant’s behaviour.

CareToy allows an intensive, individualized, home-based and family-centred intervention, managed remotely by trained clinical staff. The rehabilitative staff may use the modular organization and the possibility of activating single parts of the system (e.g. there are many lights in the wall but they are hidden and they can be variously activated associated or not with sounds/music) automatically and/or in relation to the actions of the infant (e.g. only if the infant rolls or grasps) to tailor the training to the individual needs of the infant. The infant may in this way be promoted to actively perform specific goal-directed activities (e.g. for postural control, reaching, grasping, visual attention and orientation). Feedback regarding the performance of the infant is obtained by video and sensors in toys and mattresses. They provide quantitative information about grasp shape and force, orientation and displacement in space of toys and postural changes of the infant. Acquired data are automatically uploaded to a clinical database server through the tele-rehabilitation module. This also allows the rehabilitation staff to manage the training remotely, plan new individualized exercises, assess the validity of the exercises and create and update new exercises. The CareToy training in this way guarantees a highly variable and individualized approach, tailored on the specific needs of the infant and on the achievement of specific goals.In the context of the CareToy project a platform for the quantitative evaluation of visual abilities has been also developed. This is named CareToy for Clinics and it is a highly advanced system, which is installed in the clinical centres and used only by expert rehabilitation staff. It consists of an eye tracking system (Smart Eye Pro system) equipped with 6 external infrared cameras running at 60 Hertz placed in an integrated mechanical structure with 5 screens and 5 loudspeakers (placed one in the centre, two on the right side at 30° and 60° and two on the left side, also at 30° and 60°) (Figure 1B).

To test the effects of the CareToy training on infant’s neurodevelopment we propose a randomised controlled trial (RCT) in a population of low risk preterm infants. The trial fulfils the criteria set out in the Consolidated Standards of Reporting Trials (CONSORT) Statement for Randomised Trials of non-pharmacologic treatment guidelines [26, 27].

## Methods/Design

The following hypotheses will be tested in the RCT:

-  CareToy training may improve the neurodevelopmental (e.g. motor, perceptual and cognitive) outcome in preterm infants;
-  CareToy training may help caregiver, whose role is essential for EI, in assisting skill development in their child by adapting the physical and play environment of the system;
-  CareToy system, with the characteristics pointed out above, designed and carried out in the CareToy project with also tele-rehabilitation and ICT strategies, could represent a novel home-based, rehabilitation setting for infants born preterm or at risk for neurodevelopmental disorders, for their families and for health system.

These experimental hypotheses will address the following specific aims:

- To provide first evidences that CareToy, compared to standard care, is useful to promote neurodevelopment in low risk preterm infants.
- To stimulate, by CareToy training, the caregiver-infant interaction during play activities.
- To provide evidence that the CareToy system is a suitable tool for an enriched and playful EI setting at home.
  If these aims will be achieved in low risk preterm infants, CareToy may be proposed as a new rehabilitation tool for infants at high risk of developing neurodevelopmental disorders.

### Study design

**Figure 2:**
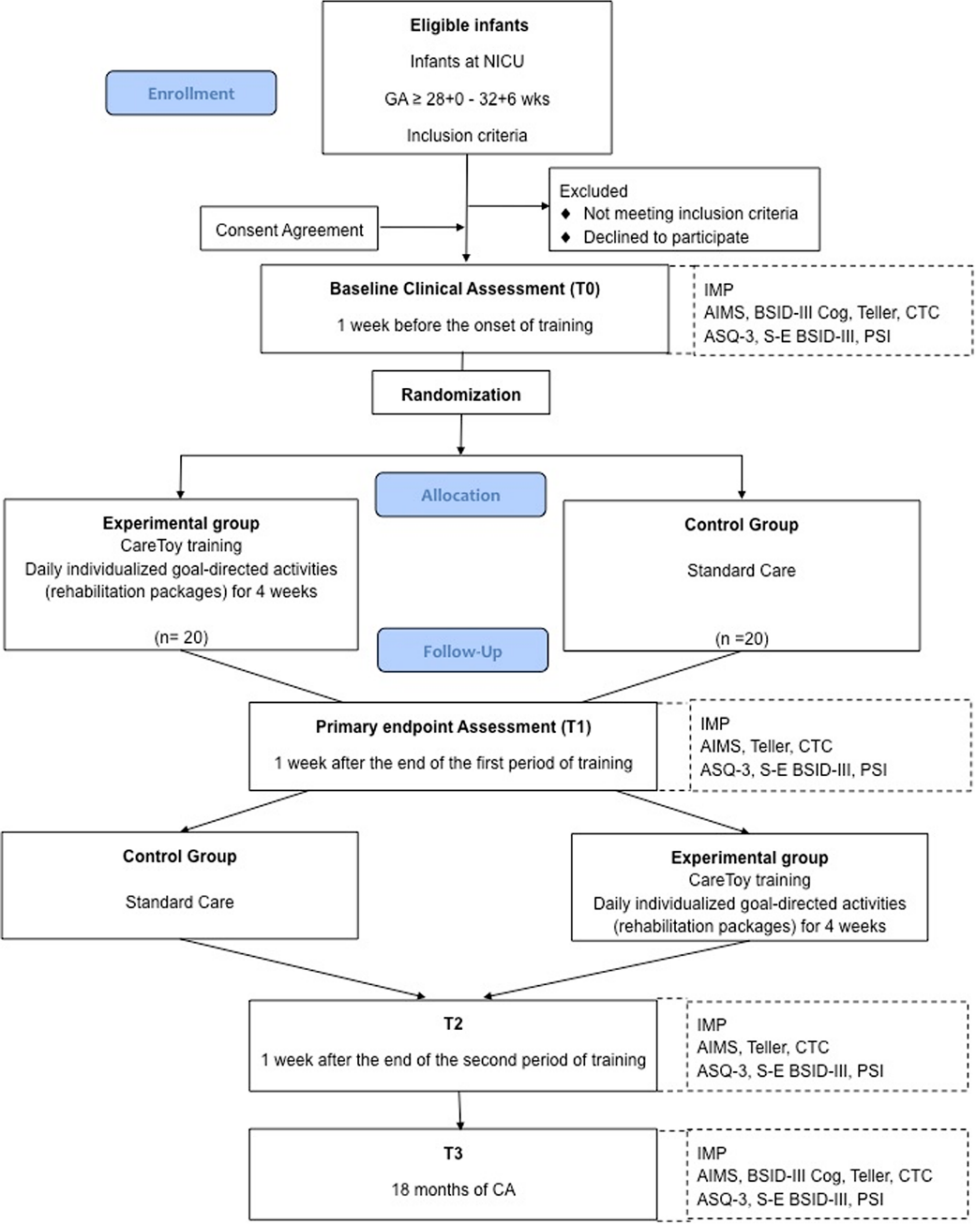
**Flow-chart of CareToy study according to CONSORT guidelines.** NICU: Neonatal Intensive Care Unit; GA: Gestational Age; IMP: Infant Motor Profile; AIMS: Alberta Infant More Scale; BSID-III Cog: Bayley Scales of Infant Development III- Cognitive Subscale; Teller: Teller Acuity Cards; CTC: CareToy for Clinics; ASQ-3: Ages & Stages Questionnaire Third Edition; S-E BSDI-III: Social-Emotional Scale of Bayley III; PSI: Parenting Stress Index; CA: Corrected Age.

A multicenter, evaluator-blinded, parallel group RCT will be carried out to compare the effects of the CareToy training to standard care in preterm infants.The two clinical centres involved in the study are the IRCCS Fondazione Stella Maris, Department of Developmental Neuroscience, in Pisa (Italy), in collaboration with Neonatal Intensive Care Unit, Pisa University Hospital “Santa Chiara” and in Copenhagen (Denmark) the Helene Elsass Center, in collaboration with the University of Copenhagen (Depts. of Neuroscience and Pharmacology and Neonatology, Rigshospitalet). Clinical assessment will be performed at baseline (T0, in the week preceding the CareToy intervention/standard care) and in the week after the end of CareToy training/standard care (T1, primary endpoint). For ethical issues, after T1, the infants who start with standard care will have the opportunity to receive the CareToy training, while the infants that have already performed CareToy training will continue standard care. At the end of this open phase (T2), all the infants will be assessed as at T1. Moreover, a further assessment will be performed at 18 months of corrected age (T3) to evaluate the long-term effects on neurodevelopmental outcome. The experimental design and the timeline are described in detail in Figure 2. A pilot study has been performed in order to assess the feasibility of the CareToy training and to tune and set-up the CareToy system and the rehabilitation packages.

### Study sample and recruitment

The study population will consist of preterm infants recruited at the local Neonatology Units (“Santa Chiara” University Hospital in Pisa and Rigshospitalet in Copenhagen). Eligible infants will be identified according to strict inclusion and exclusion criteria (see below) by the Neonatology teams before the discharge. They will inform the caregivers about the study and, if interested, the information letter and the informative flyer about the project will be given. Recruitment should take place after discharge until 9 months of corrected age. If the parents accept to participate they will provide their written informed consent for the infant to take part of the study. Extensive perinatal data will be collected from medical records and the infants will be evaluated on the basis of a standard neurological examination (Hammersmith Neurological Examination, HINE [28, 29] and by Prechtl’s General Movements Assessment, GMA [30, 31]).

The clinical trial has been approved by the Ethics Committee of Pisa University Hospital (Italy), Tuscan Region Pediatric Ethics Committee (Italy) and to Ethics Committee of Region Hovedstaden (Denmark). Moreover, the Italian Ministry of Health has approved the trial considering that the CareToy is configured as a medical device without a CE mark.

This study will include preterm infants that met the following criteria:

Inclusion Criteria:

-  gestational age ≥28 + 0 weeks and 32 + 6 weeks
-  corrected age at baseline: between 3 and 9 months;
-  achievement of predefined cut off scores in gross motor ability derived from Ages & Stages Questionnaire® Third Edition (ASQ-3), in relation to corrected age [32].

In detail:

- 4 months form (from 3 months to 4 months 30 days) score ≥10;
- 6 months form (from 5 months to 6 months 30 days) score ≥5 - <50;
- 8 months form (from 7 months to 8 months 30 days) ≥10 - <30

Exclusion Criteria:

-  infants with gestational age <28 weeks or ≥33 weeks
-  infants small for gestational age (i.e. weight below the 10^th^)
-  presence of brain damage i.e. brain malformation, intra-ventricular haemorrhage (IVH) >1; any degree of periventricular leukomalacia (PVL)
-  known epilepsy or other form of seizure
-  severe sensory deficits (blindness, deafness)
-  other severe non neurological malformations
-  participation in other experimental studies having rehabilitation aims

### Sample size

According to CONSORT guidelines [26, 27] the sample size estimates were based on projected treatment effect on the primary outcome measure, the Infant Motor Profile (IMP [33, 34], see below). A sample of 36 infants is required to detect a clinically relevant change of 7.5 points in the total IMP score (SD = 8.2) at significance level of 0.05 and 80% power [35]. Considering a 10% of possible drop-outs a minimum of 40 infants will be recruited. Twins will always be assigned to the same group, in order to facilitate the parents in managing the CareToy system for their infants.

### Randomisation

After enrolment, infants will be randomly allocated to the intervention (CareToy training) or control group using a computer generated set of random pair 1:1 allocation (CareToy training – standard care or standard care – CareToy training). These random sets will be sealed in numbered envelopes.

### Blinding

Caregivers, therapists and study personnel who follow the infants during the trial will not be blinded about the allocation group. Outcome measures (Infant Motor Profile, IMP; Alberta Infant Motor Scale, AIMS; Bayley Scales of Infant Development III Edition, BSID-III) will be performed, videotaped and scored by assessors who are blinded to group allocation.

### Therapy protocols

#### CareToy training

On the base of each infant’s developmental needs, the rehabilitation staff (e.g. child neurologists and paediatric physical therapists) will plan a series of individualized goal-directed activities (rehabilitation packages) mainly focused on postural control, reaching, grasping, manipulation, visual attention and orientation in supine, prone and/or sitting position. According to the planned activities, the CareToy system will be personalized and delivered at the home of the infant. The CareToy training will be used daily for 4 weeks (28 days). Each daily training lasts about 30-45 minutes and consists of various activities of 2-10 minutes duration performed during the day when the infant is in active state and compliant.

Daily training will be remotely monitored by the rehabilitation staff, who may modify the rehabilitation packages according to infant’s developmental needs and progress. Caregivers will fill out a questionnaire at the end of each training session to record acceptance and compliance to the activities by the infant. The drop-out criterion for each daily session is if the infant completes less than 51% of the planned scenarios.

Caregivers will be trained in playing with their infant through the CareToy system during the first training week by the rehabilitation staff. In the following 3 weeks of training, support and advice will be given, even on site if necessary.

#### Standard care

Standard care refers to the current care advice, which is similar in Pisa and Copenhagen, in the management of preterm infants in the first months of life.

### Outcome measures

During the study period infants will be assessed at baseline and then at three different time points (Figure 2).

The infants' assessment will be performed in each clinical centre, by blinded assessors, who are trained in the use of the outcome scales. To guarantee a high level of evaluation and score agreement, during the months preceding the beginning of the study, the assessors had worked together sharing assessments and scores.

The assessment of motor development has been chosen as primary outcome measure using IMP. The following secondary outcomes have been chosen: AIMS, BSID-III Cognitive subscale and Teller Acuity Cards®. Each assessment can be done in two consecutive days to ensure greater compliance by the infant.

### Primary outcome measure

#### Infant Motor Profile (IMP)

It is a new, video-based and qualitative assessment of motor behaviour in infancy, applicable in preterm and at term infants aged 3 to 18 months [33]. It consists of 80 items addressed to explore the child's motor abilities and to evaluate motor behaviour in five domains: i) variation, ii) variability (ability to select motor strategies), iii) movement fluency, iv) movement symmetry and v) motor performance.

IMP consists of spontaneously and elicited motor behaviour recording while the infants are in supine, prone, sitting, standing, walking (depending on infants' age and functional capacity), reaching, grasping, and manipulation of objects in supine and in supported sitting positions. The assessment lasts approximately 15 minutes. The total IMP score is constituted by the mean of the five domain scores. It is scored off-line on the basis of the video recordings.

This tool is intended to detect and quantify changes after intervention and it is reported as having a satisfactory intra and inter-observer reliability and a good score reliability. In particular, it has been demonstrated that the inter-observer reliability of the total IMP score yielded an ICC of 0.94 (95% CI 0.87–0.97), and the reliability of scoring the IMP domains moderate to good, with ICCs ranging from 0.69 to 0.99. Moreover a strong correlation between IMP performance domain score and AIMS has been found [33, 34]. This evaluation will be carried out at all four time-points (T0-T3), but only the change from T0 to T1 will be used as the primary outcome.

### Secondary outcome measures

#### Alberta Infant Motor Scale (AIMS)

This standardized scale examines delayed and abnormal motor development in infants overtime and it is a tool for assessment from term until 18 months of age [36]. AIMS assesses infant movement in four positions: prone, supine, sitting and standing, requiring about 20-30 minutes of administration time. The scale is quick to administer with limited handling and focuses on achievement of motor milestones, quality of posture and movement outcomes [37].

AIMS has been shown to be sufficiently sensitive to differentiate the motor development of preterm infants from that of full term infants. This evaluation will be carried out at all four time-points (T0-T3).

### BSID-III-Cognitive subscale

This scale is sensitive in detecting differences between a normative sample and children at risk for delayed development, such as premature infants. It has normative value referenced assessments, with means of 100 and Standard Deviation (SD) of 15 points. Children whose scores are 2 SDs below the normative value in a domain are considered as having a significant delay in that aspect of development. Bayley III is appropriate for administration to children between the ages of 1 month and 42 months (although norms extend downward to age of 16 days). The Bayley III revision includes Cognitive, Language, Motor, Social-Emotional, and Adaptive Behaviour scales. Items on the cognitive subscale assess sensor-motor development, exploration, manipulation, object relatedness, concept formation, problem-solving and memory [38]. This evaluation will be carried out at T0 and at T3.

### Teller acuity cards®

Teller Acuity Cards II is a test used to evaluate visual acuity in infants and children. It is based on judgement of the infant attention to a series of cards showing stripes of different widths. This tool allows rapid assessment of resolution (grating) visual acuity in infants and young children, and other populations where verbal response to recognition (letter) visual acuity charts is difficult or impossible [39–41]. It evaluates development of visual acuity and it has been used in several studies for diagnostic purposes and to measure the results of early intervention [18, 42, 43]. This evaluation will be carried out at all four time points (T0-T3). Visual Acuity assessment will complemented by quantitative evaluation of visual fixation and visual following, by means of an eye-tracker (CareToy for Clinics).

### Questionnaires

#### Ages & stages questionnaire® third edition (ASQ-3)

It has been developed as a screening tool for developmental delay in infants in numerous paediatric populations with reported easy administration, short completion time, easy interpretation, sensitivity, measure of true positives, and specificity, measure of true negatives, varying, with most studies reporting higher accuracy in at risk populations [32, 44–46].

The questionnaire covers from 1 to 66 months of age. The parents answer 30 questions that assess 5 domains of development, including communication, gross motor, fine motor, problem-solving, and adaptive skills. This self-administered assessment can be completed in 10 to 20 minutes and scored in 1 to 5 minutes.

The questionnaire will be given to parents after enrolment in order to define the most appropriate starting time for each infant (see inclusion criteria), Moreover, it will be given to the parents during all assessments in order to explore infant development from the parental point of view.

#### Social-emotional scale of BSID-III

It is a screening tool to measure social-emotional milestones in young children aged 0-42 months for early identification of social-emotional deficits and planning of the most successful interventions. It is filled out by the parents and contains 35 items, rated using a 5-point scale and ordered developmentally, according to age at which each item is typically mastered [47]. This questionnaire will be carried out at all time points (T0-T3).

#### Parenting Stress Index (PSI)

It is a self-report questionnaire designed to identify specific parental, child and situational characteristics most commonly associated with dysfunctional parenting [48]. Currently, the PSI is mostly used as a screening instrument for the early identification of parent-child systems, which are under stress and at risk of developing dysfunctional parenting behaviour. The validity of the PSI has been established in numerous studies on children with developmental problems, behaviour problems, disabilities and illnesses, as well as studies of at risk families and cross-cultural studies [49]. The questionnaire will be carried out at all time points (T0-T3).

### Analyses

Clinical Data will be managed and analysed using the Statistical Package for Social Sciences (SPSS). Descriptive statistics (means, standard deviation) will be calculated to summarise the data set for both groups and to identify potential baseline differences between the groups; p values will be used to indicate the strength of the evidence, and the Bonferroni correction for multiple assessments will be performed. The primary level of analysis will be the assessment of the effects of CareToy training at the primary endpoint (T1) in the primary and then in the secondary outcome measures. In the second level analysis we will provide multivariate statistics in order to take into account effect modifiers such as basal level of motor development, family compliance and time of training.

## Discussion

The paper presents the background and the design for a RCT comparing a new intervention, CareToy Home, with the standard care for infants at low risk for neurodevelopmental disorders.

The study is the first to test this new type of treatment as a new tool for an intensive, individualized, home-based and family-centred intervention, managed remotely by clinical staff. The present research aims to assess the feasibility of the system and determine its effect on the neurodevelopment of a group of preterm infants at low risk for neurodevelopmental disorders. If successful, a future aim is to apply the system in further studies involving infants at higher risk for brain lesion or genetic disorders. The CareToy Home system represents a challenging and innovative tool in the field of infant rehabilitation.

This trial will explore the applicability of a new frontier of tele-medicine in infants. This opens the possibility of managing early intervention in infants at home from the clinical centre, expanding the access of infants to EI. Moreover, the rigorous scientific design, according to CONSORT guidelines [26, 27] will allow us to evaluate the effects of CareToy training on neuro-developmental outcome in a first sample of at low risk preterm infants.

## Authors’ information

GS is specialist in child neuropsychiatry and researcher at IRCCS Fondazione Stella Maris in Pisa, which is a research hospital for children and adolescents with neurological and psychiatric disorders, located in Calambrone, Pisa. LB is senior clinical register at Pisa University Hospital (AOUP), Neonatology Unit and responsible of follow-up program. GC is specialist in child neuropsychiatry, full professor of child neuropsychiatry at University of Pisa and Scientific Director of IRCCS Fondazione Stella Maris. GG is consultant neonatologist of the Department of Neonatology, Rigshospitalet, professor of paediatrics, and chair of paediatrics at the Institute of Clinical Medicine, University of Copenhagen. AH is MD and PhD student at the Health Science Faculty, University of Copenhagen where she is associated to the Department of Neuroscience and Pharmacology and to the Department of Neonatology. EI is specialist in child neuropsychiatry and PhD student in neuroscience at Scuola Superiore Sant’Anna, Pisa. JL is research supervisor at the Helene Elsass Center and Research Associate Professor at the Department of Nutrition, Exercise and Sport, University of Copenhagen. JBN is full professor of Neuroscience and head of the research section, Neural Control of Movement, at the Department of Neuroscience and Pharmacology and the Department of Nutrition, Exercise and Sport, University of Copenhagen. ES is senior paediatric physical therapist at IRCCS Fondazione Stella Maris.

## Abbreviations

AIMS: Alberta infant motor scale
ASQ-3: Ages & stages questionnaire – 3
BSID-III: Bayley scales of infant development – III
CI: Confidence interval
CONSORT: Consolidated standards of reporting trials
EE: Enriched environment
EI: Early intervention
EU: European Union
EC: European Conformity
GMA: General Movement Assessment
HINE: Hammersmith neurological examination
ICT: Information and communication technologies
IMP: Infant motor profile
IVH: Intra-ventricular Haemorrhage
NICU: Neonatology Intensive Care Unit
PSI: Parenting stress index
PVL: Periventricular leukomalacia
RCT: Randomised controlled trial
SD: Standard deviation
SPSS: Statistical package for social sciences
VLBW: Very low birth weight.
